# Practical Notes on Diagnosis and Treatment

**Published:** 1907-11-23

**Authors:** 


### Practical Notes on Diagnosis and Treatment.

### Cardinal Rules in Diagnosis.

The first rule is to examine the whole patient; the second the whole of the diseased part; the third to compare opposite sides of the body; and the fourth to examine the diseased part at rest.—*Mr. Lockwood.*

### Post-diphtheritic Ocular Palsies.

Diphtheritic paralysis of the ciliary muscle and of the sphincter iridis is not very uncommon. Paralysis of the external ocular muscles in this disease is much less frequent, but it does sometimes occur.

### Duodenal Ulcer.

This is more common in men than women. Pain coming on two to three hours after food and in the anatomical situation of the pylorus is suggestive. The stools should be examined, as melæna is a common result. Progressive anæmia is sometimes explained in this way. The altered appearance of the blood prevents the patient recognising the fact of hæmorrhage.

### Chorea Gravidarum.

In the treatment of chorea in pregnancy the first thing to do is to try to get the patient to sleep and to see that she is taking proper food. If it is a mild attack it will gradually wear itself out. Probably the best drug in the mild cases, where it simply wants a sedative, is bromide, or if sleep is wanting, bromide and chloral. But in severe cases, if you find that drugs fail to act, you will have to empty the uterus, trying digital dilatation first.—*Dr. Amand Routh.*

### Oil of Cajuput.

Three drops of oil of cajuput on a piece of sugar or on crumb of bread taken frequently is worth all the other antifermentatives put together.—*Dr. Murrell.*

### Quinine in Leucorrhœa.

Hydrochlorate of quinine, one grain to the ounce, dissolved in warm boric acid solution, is an excellent douche in cases of leucorrhœa. The salt may also be prescribed in the form of pessaries, two or three grains in each. The pessaries may be made with a glyco-gelatine basis, and in this hazeline may be used instead of water.

### Paraldehyde in Asthma.

Potassium iodide and tincture of lobelia are much more successful in relieving the asthmatic paroxysm if the patient is able to sleep. To secure this there is nothing better than paraldehyde. It should be given in a dose of one drachm, and its disagreeable taste may be disguised by syrup of orange and cinnamon water.

### Lavage of the Intestine.

Two or three times a day introduce by a long soft tube three and a half pints of boiled water. A saline, such as potassium acetate, may be added. This is a useful measure to promote diuresis, especially when uræmia is threatening. In the same circumstances lavage of the stomach may be practised and saline fluid introduced into the subcutaneous tissue.

